# Supplementary material for: Genetic Elucidation for Response of Flowering Time to Ambient Temperatures in Asian Rice Cultivars
Source: Int J Mol Sci. 2021 Jan 20;22(3):1024. doi: 10.3390/ijms22031024 (PMC7864171; doi:10.3390/ijms22031024)
Supplement: Supplementary file 1 [file ijms-22-01024-s001.pdf]

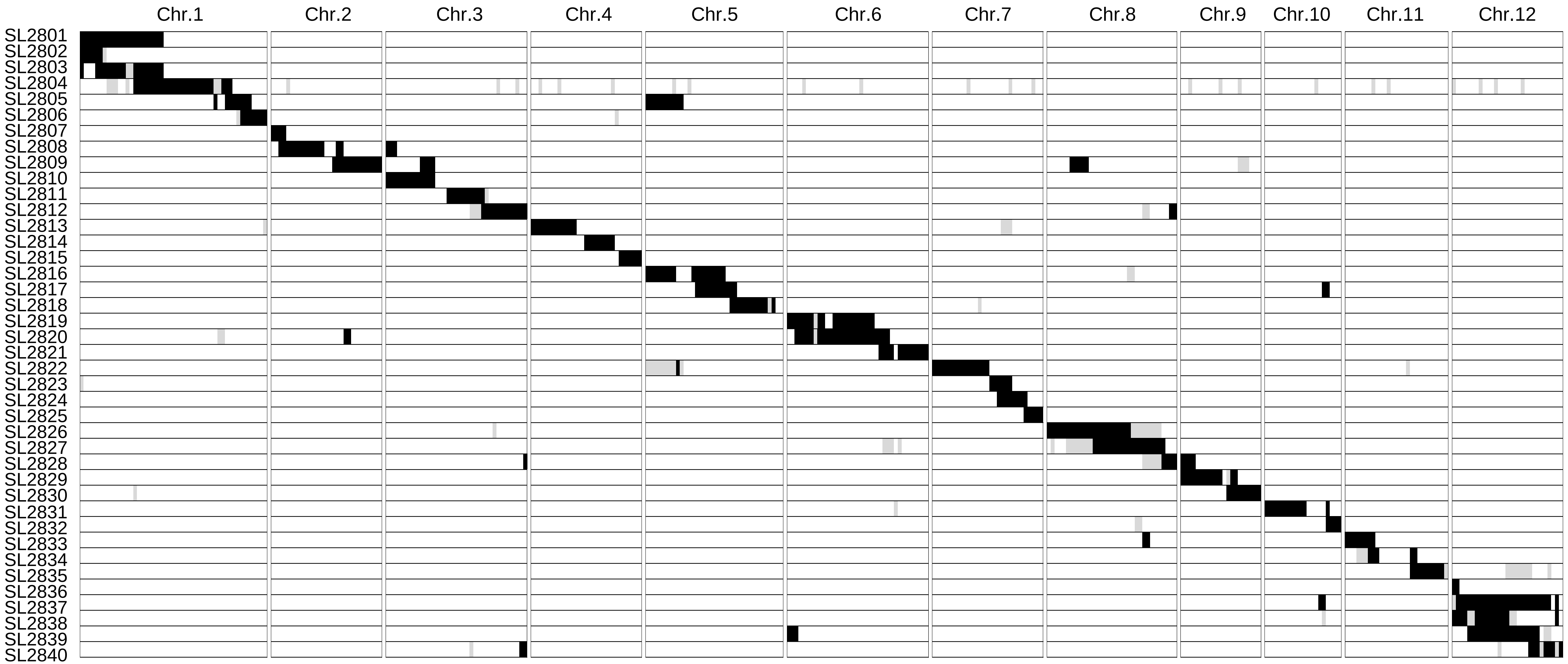

Supplementary Figure S1. Graphical representation of genotypes of 40 chromosome segment substitution lines (CSSLs) with introgressed chromosome segments from ‘Khao Nam Jen’ in the genetic background of ‘Koshihikari’. Each chromosome is oriented from the short arm (left) to the long arm (right). Black, homozygous for ‘Khao Nam Jen’; white, homozygous for ‘Koshihikari’; gray, heterozygous. These genotypes are taken from [27].

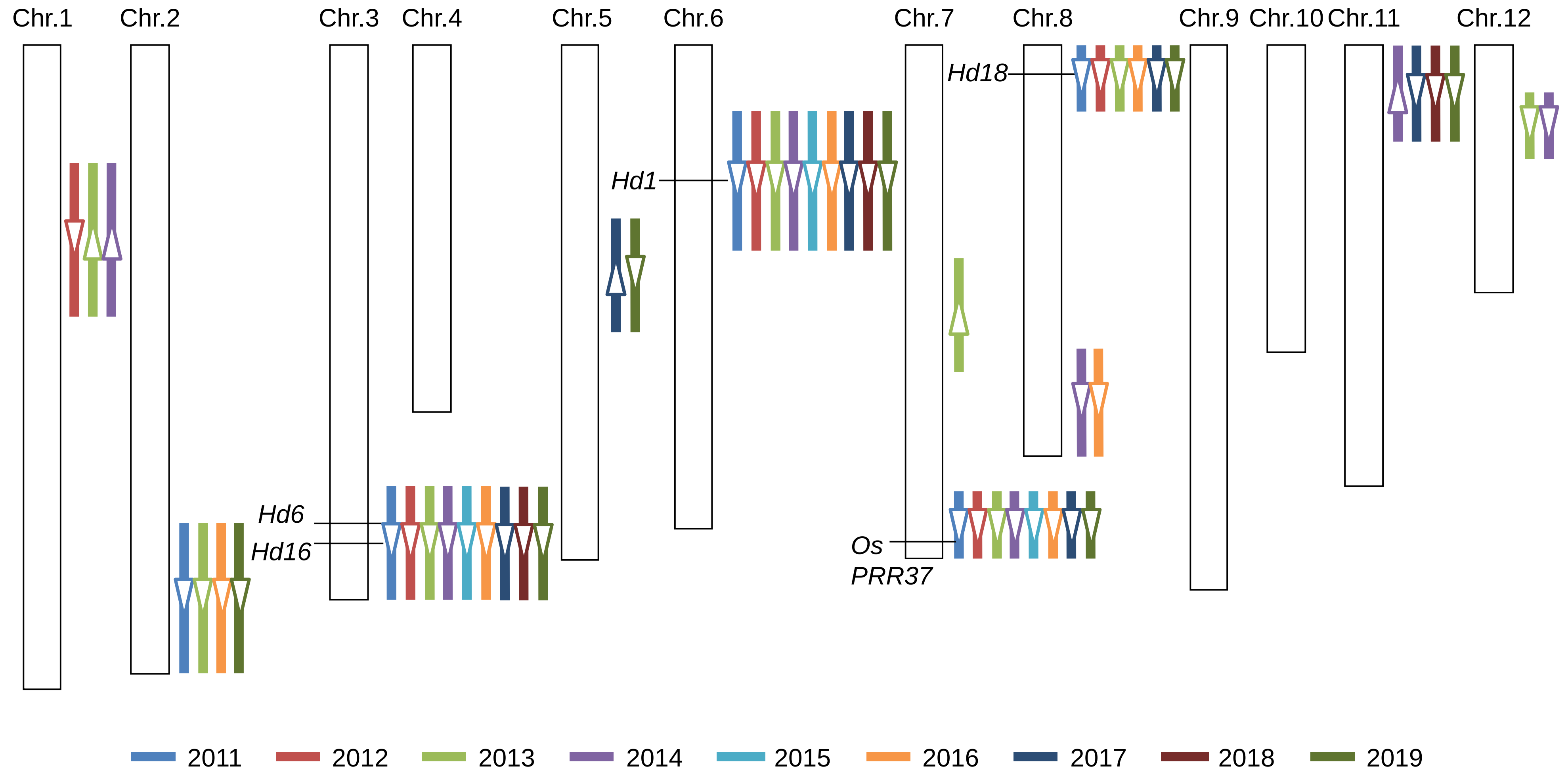

Supplementary Figure S2. Chromosome positions of QTLs for flowering time in each year in CSSLs. Boxes indicate the 12 rice chromosomes. Vertical bars on the right side of boxes indicate positions of QTLs causing difference of flowering time from 'Koshihikari' and the introgressed chromosome segment from 'Khao Nam Jen' in each CSSL. Downward and upward triangles indicate direction of genetic effect of 'Khao Nam Jen' alleles: ▽ late flowering; △ early flowering. The five flowering-time genes with allelic differences between 'Khao Nam Jen' and 'Koshihikari' shown on chromosomes 3, 6, 7, and 8 are cited from [30, 31].

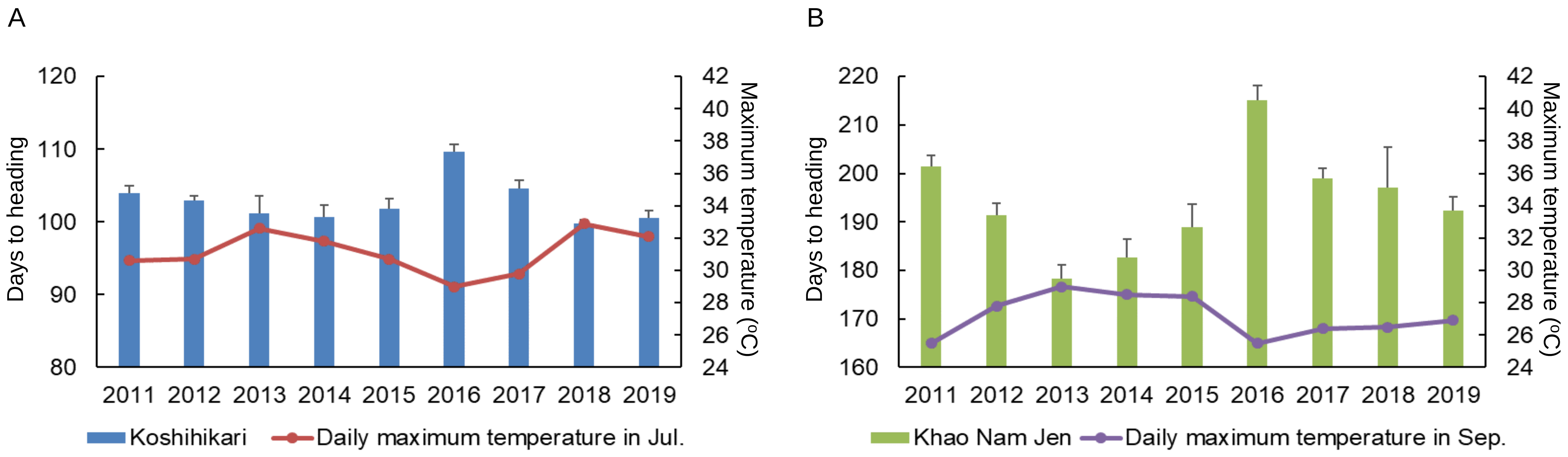

Supplementary Figure S3. Days-to-heading date and daily maximum temperatures at panicle initiation stage in (A) ‘Koshihikari’ (July) and (B) ‘Khao Nam Jen’ (September) from 2011 to 2019.

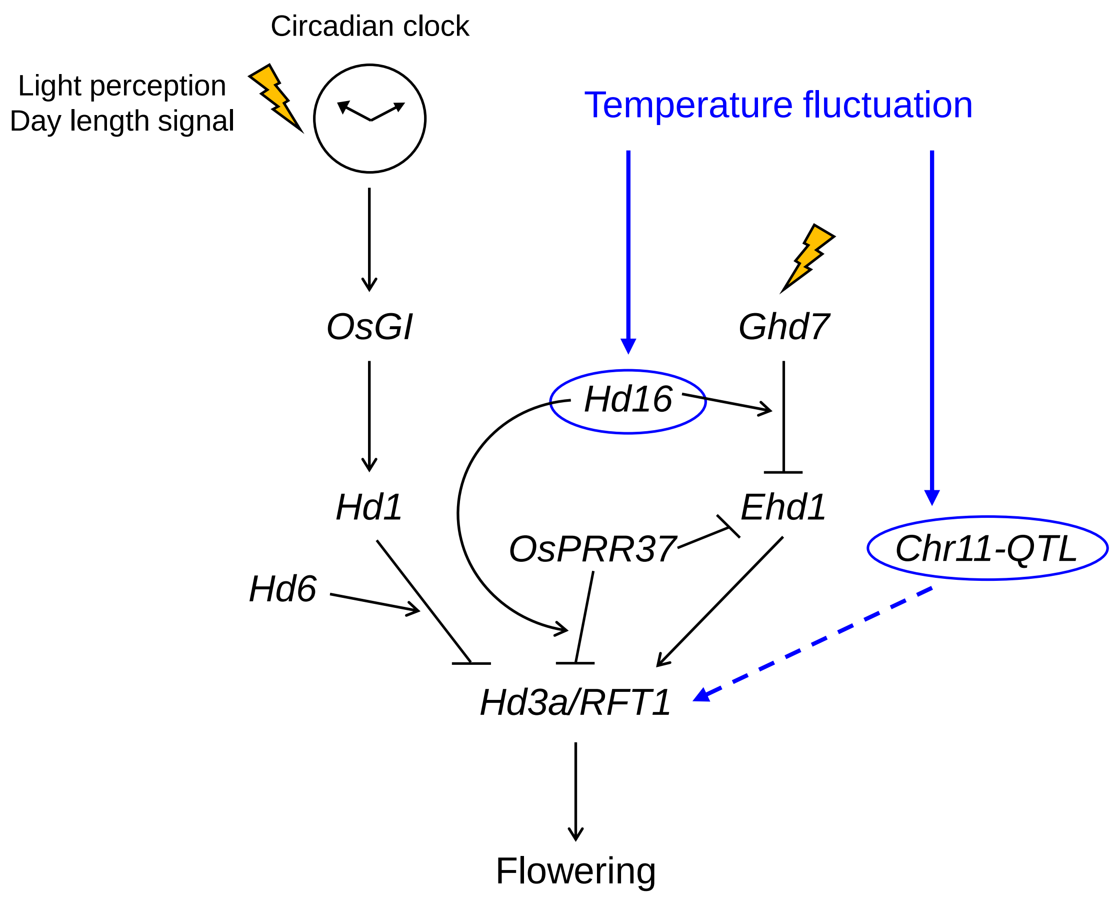

Supplementary Figure S4. Genetic pathway controlling flowering time in rice under long daylength with ambient temperature fluctuations. ↓ Upregulation; ⊥ downregulation.

Supplementary Table S1. Days-to-heading date in 18 Asian rice cultivars, including ‘Koshihikari’ and ‘Khao Nam Jen’, 40 CSSLs with ‘Khao Nam Jen’ chromosome segments in the ‘Koshihikari’ genetic background, and two ‘Koshihikari’ NILs with introgressed *Hd6* and *Hd16* genes, in 2011–2019.

		2011	2012	2013	2014	2015	2016	2017	2018	2019	Difference of days-to-heading among years	Coefficient of variation
Qui Zhao Zong	*indica*	87.6 ± 1.1	89.4 ± 0.5	86.0 ± 0.7	81.8 ± 1.1	84.8 ± 0.8	87.4 ± 2.3	87.0 ± 1.6	82.8 ± 1.5	88.4 ± 2.3	7.6	3.0
Tupa 121-3	*aus*	104.2 ± 0.8	101.2 ± 0.4	96.0 ± 1.2	95.2 ± 0.4	91.8 ± 1.1	106.4 ± 1.3	101.4 ± 2.8	92.4 ± 0.9	100.0 ± 2.6	14.6	5.2
Kasalath	*aus*	107.0 ± 0.7	105.2 ± 1.3	101.8 ± 0.8	97.6 ± 1.1	99.2 ± 0.4	109.0 ± 0.7	109.4 ± 2.3	99.4 ± 0.5	102.6 ± 0.5	11.8	4.2
Muha	*aus*	111.0 ± 1.9	105.6 ± 1.7	100.8 ± 0.8	98.4 ± 1.1	97.0 ± 0.7	110.4 ± 0.5	109.8 ± 1.5	96.8 ± 2.4	100.8 ± 0.8	14.2	5.7
Davao1	*indica*	109.2 ± 0.8	108.8 ± 0.4	106.0 ± 1.0	103.0 ± 1.0	100.8 ± 0.8	109.8 ± 1.3	107.4 ± 1.5	97.8 ± 0.4	106.8 ± 1.3	12.0	3.9
Nipponbare	*japonica*	112.2 ± 1.3	109.2 ± 1.9	109.6 ± 0.5	105.8 ± 0.8	107.4 ± 1.3	116.0 ± 1.2	114.4 ± 0.5	105.2 ± 0.8	106.2 ± 1.3	10.8	3.5
IR64	*indica*	113.8 ± 0.8	112.4 ± 1.1	110.6 ± 1.5	107.6 ± 0.9	106.0 ± 0.7	118.2 ± 1.1	117.4 ± 0.9	109.4 ± 0.5	108.4 ± 0.9	12.2	3.8
Touboshi	*indica*	115.6 ± 1.1	115.0 ± 2.3	110.4 ± 2.1	105.4 ± 0.9	104.8 ± 1.3	116.2 ± 1.8	113.6 ± 0.5	104.6 ± 1.3	103.6 ± 0.9	12.6	4.8
Basilanon	*aus*	111.8 ± 1.3	116.6 ± 2.9	114.4 ± 2.3	108.4 ± 0.9	111.0 ± 1.0	115.4 ± 2.3	112.4 ± 0.5	106.8 ± 1.1	111.8 ± 1.3	9.8	2.8
Deng Pao Zhai	*indica*	119.8 ± 1.9	114.3 ± 1.0	117.0 ± 1.4	117.4 ± 1.1	110.6 ± 0.5	123.4 ± 2.4	115.0 ± 2.0	109.2 ± 0.8	113.0 ± 2.5	14.2	3.9
Khau Mac Kho	*japonica*	119.2 ± 0.4	122.0 ± 1.0	118.8 ± 1.5	117.4 ± 1.9	113.0 ± 2.5	125.8 ± 1.5	129.2 ± 1.1	114.6 ± 2.4	115.4 ± 2.1	16.2	4.5
LAC23	*japonica*	125.2 ± 3.1	125.4 ± 1.1	125.2 ± 1.3	123.2 ± 0.8	117.8 ± 1.6	127.0 ± 0.7	126.4 ± 1.7	119.2 ± 1.1	121.2 ± 1.9	9.2	2.7
Naba	*indica*	128.0 ± 1.9	125.4 ± 1.1	124.6 ± 1.1	123.8 ± 1.3	118.8 ± 0.8	131.2 ± 0.8	131.4 ± 1.1	121.8 ± 1.1	120.8 ± 1.9	12.6	3.5
Bei Khe	*indica*	136.0 ± 0.7	125.2 ± 1.3	124.6 ± 1.1	125.2 ± 1.6	112.8 ± 1.8	128.8 ± 1.3	126.0 ± 1.4	121.4 ± 3.7	116.4 ± 2.2	23.2	5.4
Bleiyo	*indica*	188.0 ± 1.0	189.2 ± 0.4	190.6 ± 2.2	186.3 ± 1.5	191.6 ± 0.5	187.4 ± 1.3	190.3 ± 4.0	185.8 ± 0.8	182.6 ± 1.3	9.0	1.5
Nona Bokra	*indica*		199.6 ± 1.3	209.2 ± 1.3	200.8 ± 1.3	215.0 ± 0.5	194.4 ± 0.5	205.6 ± 0.5	196.0 ± 1.4	215.0 ± 0.5	20.6	3.9
Koshihikari	*japonica*	103.9 ± 1.0	102.9 ± 0.7	101.2 ± 2.3	100.6 ± 1.7	101.8 ± 1.3	109.7 ± 0.9	104.6 ± 1.1	99.8 ± 0.4	100.6 ± 0.9	9.9	3.0
Khao Nam Jen	*japonica*	201.4 ± 2.3	191.3 ± 2.5	178.3 ± 2.8	182.6 ± 3.8	189.0 ± 0.0	215.0 ± 4.7	199.0 ± 0.0	197.0 ± 23.7	192.3 ± 2.5	36.7	5.6
SL2801		106.2 ± 2.3	104.4 ± 1.1	100.4 ± 2.7	100.4 ± 1.8	102.6 ± 1.7	108.8 ± 0.8	102.4 ± 1.3	99.0 ± 1.4	100.4 ± 1.7	9.8	3.1
SL2802		105.2 ± 2.9	103.0 ± 0.7	100.8 ± 1.6	98.8 ± 0.8	101.2 ± 1.1	110.6 ± 0.9	102.6 ± 2.9	99.0 ± 1.4	101.4 ± 1.3	11.8	3.5
SL2803		103.8 ± 2.8	100.8 ± 0.8	94.2 ± 1.5	96.8 ± 0.4	100.6 ± 1.1	108.0 ± 1.0	103.0 ± 1.2	97.4 ± 1.7	100.4 ± 1.8	13.8	4.1
SL2804		109.4 ± 1.5	105.4 ± 1.1	100.8 ± 2.2	101.4 ± 0.9	102.4 ± 1.1	111.4 ± 1.1	104.8 ± 0.8	95.6 ± 0.9	102.0 ± 0.7	15.8	4.6
SL2805		104.0 ± 1.4	104.0 ± 0.7	99.6 ± 2.1	99.0 ± 1.4	101.4 ± 0.9	111.0 ± 1.2	107.2 ± 1.8	101.0 ± 2.2	101.4 ± 1.1	12.0	3.8
SL2806		103.4 ± 0.9	103.2 ± 0.8	100.0 ± 2.0	97.8 ± 1.5	99.8 ± 1.6	110.2 ± 0.8	104.4 ± 0.9	99.8 ± 1.5	101.0 ± 0.7	12.4	3.6
SL2807		104.8 ± 0.8	103.8 ± 0.4	99.4 ± 3.0	97.4 ± 2.4	101.0 ± 1.0	110.4 ± 0.9	104.4 ± 0.5	97.4 ± 1.1	101.0 ± 0.7	13.0	4.1
SL2808		106.4 ± 1.8	103.4 ± 0.5	100.6 ± 1.8	100.0 ± 1.2	100.4 ± 0.9	111.0 ± 0.7	106.2 ± 2.2	99.2 ± 0.8	101.6 ± 1.1	11.8	3.8
SL2809		120.9 ± 6.3	105.6 ± 0.9	104.2 ± 1.1	103.6 ± 1.8	103.6 ± 0.5	114.2 ± 0.4	107.2 ± 2.2	99.0 ± 0.7	103.0 ± 1.2	18.9	6.3
SL2810		107.4 ± 1.3	105.2 ± 0.8	102.6 ± 0.5	101.6 ± 1.3	101.0 ± 1.4	111.8 ± 1.3	105.6 ± 1.5	102.0 ± 2.0	101.0 ± 0.7	10.8	3.5
SL2811		108.8 ± 1.6	104.4 ± 0.5	101.6 ± 0.5	96.6 ± 1.1	100.2 ± 1.5	110.0 ± 0.7	104.2 ± 2.3	100.6 ± 0.9	102.2 ± 0.8	13.4	4.1
SL2812		124.4 ± 2.3	123.2 ± 0.7	123.0 ± 0.9	115.2 ± 2.1	105.4 ± 1.5	128.4 ± 0.9	132.8 ± 1.6	120.8 ± 0.4	119.4 ± 1.7	23.4	6.5
SL2813		106.0 ± 1.6	103.2 ± 0.8	96.6 ± 1.5	97.2 ± 1.5	99.4 ± 0.9	109.4 ± 2.1	101.2 ± 1.3	98.8 ± 1.8	101.0 ± 1.2	12.8	4.1
SL2814		105.6 ± 2.2	104.0 ± 1.0	100.4 ± 0.9	98.8 ± 1.8	100.8 ± 1.3	109.6 ± 0.5	101.0 ± 1.0	97.4 ± 1.3	101.0 ± 1.2	12.2	3.7
SL2815		105.6 ± 2.1	105.6 ± 0.9	102.0 ± 0.7	100.4 ± 0.5	102.0 ± 0.7	111.0 ± 1.2	105.4 ± 3.1	98.6 ± 0.9	101.8 ± 1.1	12.4	3.6
SL2816		106.2 ± 1.5	105.6 ± 1.1	102.4 ± 0.9	101.2 ± 0.8	102.4 ± 0.5	111.0 ± 1.2	104.6 ± 2.4	99.6 ± 1.5	101.4 ± 2.1	11.4	3.3
SL2817		105.0 ± 2.0	103.2 ± 1.6	99.4 ± 1.5	98.2 ± 1.6	101.2 ± 0.8	109.8 ± 0.8	100.6 ± 0.9	99.8 ± 0.8	103.2 ± 1.5	11.6	3.5
SL2818		107.2 ± 1.9	106.4 ± 1.1	101.4 ± 1.5	98.0 ± 1.6	102.0 ± 1.4	110.8 ± 1.3	108.0 ± 1.2	101.2 ± 1.6	101.0 ± 1.2	12.8	4.0
SL2819		121.2 ± 1.1	116.4 ± 1.8	117.6 ± 0.5	117.4 ± 1.8	112.4 ± 1.7	124.4 ± 1.1	125.2 ± 1.1	119.0 ± 1.0	115.8 ± 1.1	12.8	3.5
SL2820		122.2 ± 1.3	117.8 ± 0.8	117.2 ± 0.8	118.2 ± 1.1	113.2 ± 1.3	126.0 ± 0.7	125.6 ± 2.5	119.2 ± 1.9	116.0 ± 1.0	12.8	3.6
SL2821		105.7 ± 2.2	105.8 ± 0.4	101.2 ± 1.1	100.4 ± 1.5	102.0 ± 1.4	111.4 ± 0.9	106.6 ± 1.7	118.8 ± 0.8	101.4 ± 0.9	18.4	5.6
SL2822		106.0 ± 3.1	104.2 ± 0.8	103.4 ± 0.9	100.4 ± 2.1	101.2 ± 1.6	111.8 ± 0.8	104.2 ± 3.0	100.8 ± 0.8	99.8 ± 1.1	12.0	3.6
SL2823		106.6 ± 2.2	104.0 ± 0.0	100.0 ± 0.8	98.0 ± 1.2	100.2 ± 0.8	110.0 ± 1.2	103.6 ± 2.1	101.2 ± 0.8	101.6 ± 0.9	12.0	3.6
SL2824		106.8 ± 0.4	104.4 ± 1.1	101.6 ± 2.1	99.0 ± 0.7	101.4 ± 1.3	111.0 ± 0.7	104.8 ± 0.8	99.4 ± 0.9	100.0 ± 1.2	12.0	3.8
SL2825		110.4 ± 0.5	108.6 ± 0.5	107.0 ± 1.2	104.2 ± 0.8	104.6 ± 1.7	115.6 ± 0.9	111.6 ± 1.5	98.6 ± 0.5	105.8 ± 1.1	17.0	4.6
SL2826		110.2 ± 1.1	108.0 ± 0.0	105.0 ± 1.9	103.0 ± 0.7	104.0 ± 0.0	114.0 ± 0.7	108.8 ± 1.6	104.6 ± 1.5	104.0 ± 1.0	11.0	3.4
SL2827		105.4 ± 2.7	103.4 ± 1.1	99.2 ± 1.1	98.0 ± 2.0	100.2 ± 1.5	109.6 ± 0.9	106.2 ± 1.8	102.2 ± 1.3	101.6 ± 1.3	11.6	3.6
SL2828		107.2 ± 2.2	104.4 ± 0.5	99.8 ± 1.6	99.8 ± 1.1	101.8 ± 0.4	112.0 ± 1.2	104.4 ± 0.9	99.0 ± 1.2	101.4 ± 0.9	13.0	4.1
SL2829		103.8 ± 1.8	104.2 ± 1.1	100.4 ± 1.3	100.2 ± 0.8	101.4 ± 0.9	110.4 ± 0.9	106.2 ± 1.3	99.8 ± 0.8	101.6 ± 0.9	10.6	3.4
SL2830		103.4 ± 1.8	104.0 ± 0.7	98.6 ± 1.1	97.8 ± 1.6	99.6 ± 1.1	109.4 ± 0.9	103.4 ± 1.1	99.8 ± 0.8	99.2 ± 0.8	11.6	3.6
SL2831		106.2 ± 1.5	104.2 ± 0.8	102.8 ± 1.6	100.0 ± 1.2	101.8 ± 0.8	110.2 ± 0.8	106.2 ± 1.3	98.2 ± 0.8	100.4 ± 0.5	12.0	3.6
SL2832		102.8 ± 1.9	103.0 ± 1.2	100.4 ± 0.5	98.4 ± 1.3	99.8 ± 0.4	108.6 ± 1.1	101.0 ± 2.2	99.2 ± 1.1	100.2 ± 1.5	10.2	3.0
SL2833		107.2 ± 1.6	105.2 ± 0.4	101.2 ± 1.3	96.8 ± 1.9	100.8 ± 0.8	122.2 ± 0.8	124.8 ± 1.6	97.6 ± 0.5	112.8 ± 0.4	28.0	9.6
SL2834		105.2 ± 2.3	104.0 ± 1.4	98.6 ± 2.4	96.2 ± 1.1	100.4 ± 0.9	109.8 ± 1.1	104.4 ± 2.5	113.8 ± 1.3	101.4 ± 1.1	17.6	5.3
SL2835		105.2 ± 1.5	105.6 ± 0.5	100.8 ± 0.8	99.2 ± 1.3	102.6 ± 0.9	111.4 ± 0.9	106.6 ± 1.1	99.8 ± 1.3	100.4 ± 1.3	12.2	3.9
SL2836		104.8 ± 1.3	105.0 ± 1.2	102.4 ± 1.1	98.4 ± 0.5	100.2 ± 1.6	111.2 ± 1.6	102.8 ± 0.8	100.6 ± 1.1	99.8 ± 0.8	12.8	3.8
SL2837		106.2 ± 0.4	105.4 ± 0.9	104.4 ± 1.1	100.4 ± 1.5	101.8 ± 2.0	111.2 ± 1.3	107.6 ± 2.3	98.6 ± 0.5	101.2 ± 1.3	12.6	3.8
SL2838		105.8 ± 1.1	106.0 ± 1.0	104.8 ± 1.3	99.8 ± 1.3	102.8 ± 1.1	110.6 ± 0.5	106.2 ± 2.5	100.8 ± 0.8	100.0 ± 1.0	10.8	3.4
SL2839		106.2 ± 1.1	105.6 ± 0.5	102.4 ± 1.5	100.6 ± 0.9	101.4 ± 0.9	111.0 ± 0.7	105.2 ± 2.6	100.2 ± 0.4	100.8 ± 1.1	10.8	3.5
SL2840		105.4 ± 3.0	103.8 ± 0.8	99.2 ± 2.5	97.0 ± 1.4	99.8 ± 1.8	109.0 ± 0.7	103.2 ± 3.4	100.0 ± 1.0	98.4 ± 1.1	12.0	3.8
Koshihikari *Hd6* -NIL		116.0 ± 0.7	110.2 ± 0.8	111.8 ± 1.3	107.8 ± 0.8	108.0 ± 1.0	115.2 ± 1.5	118.6 ± 1.3	110.6 ± 1.1	108.8 ± 1.1	10.8	3.4
Koshihikari *Hd16* -NIL		115.0 ± 1.6	112.2 ± 0.4	112.0 ± 1.0	109.8 ± 0.7	103.8 ± 0.4	122.2 ± 0.8	125.4 ± 0.9	115.2 ± 2.4	111.6 ± 1.1	21.6	5.7

Supplementary Table S2. Loci of genes for phytochrome, cryptochrome, and auxin responses involved in flowering time regulation in response to ambient temperature in the RAP-DB.

Gene ID	Description	Position	Gene Symbol Synonym
Os01g0169800	Tryptophan aminotransferase, Indole-3-acetic acid (IAA) biosynthesis (Os01t0169800-01)	chr01:3577473..3581859	FIB, fib
Os01g0178500	A member of rice Aux/IAA family, Cross-talk of auxin and brassinosteroid signaling pathways, Plant morphogenesis (Os01t0178500-02)	chr01:4073916..4076438	OsIAA1
Os01g0182600	Orthologue of the Arabidopsis GIGANTEA, Regulation of circadian rhythm and flowering time, Photoperiodic control of flowering, Osmotic stress response (Os01t0182600-01);GIGANTEA protein. (Os01t0182600-03)	chr01:4329362..4338486	GI, OsGI
Os01g0190300	Similar to Auxin-responsive protein IAA26 (Indoleacetic acid-induced protein 26) (Phytochrome-associated protein 1). (Os01t0190300-01)	chr01:4816831..4818958	
Os01g0202700	Similar to Flowering locus T3. (Os01t0202700-00)	chr01:5652593..5655352	
Os01g0218500	Similar to ZCN14 protein. (Os01t0218500-01);FLOWERING LOCUS T (FT)-Like homolog, FT-Like1 homologous to Arabidopsis Flowering Locus T gene, Promotion of flowering (Os01t0218500-02);Similar to ZCN14 protein. (Os01t0218500-03);Non-protein coding transcript. (Os01t0218500-04)	chr01:6494446..6499766	OsFTL1
Os01g0231000	Similar to Auxin-responsive protein (Aux/IAA) (Fragment). (Os01t0231000-01);Similar to Auxin-responsive protein (Aux/IAA) (Fragment). (Os01t0231000-02);Similar to Auxin-responsive protein (Aux/IAA) (Fragment). (Os01t0231000-03)	chr01:7249771..7254629	
Os01g0277500	Dof (DNA binding with one finger) transcription factor, C2-C2 zinc finger transcription factor, Regulation of flowering time (Os01t0277500-01)	chr01:9733832..9738224	OsDof4
Os01g0286100	Phytochrome-interacting factor-like protein, Basic helix-loop-helix factor, Repression of seedling growth in the dark (Os01t0286100-01)	chr01:10271157..10274304	PIL15, OsPIL15, OsbHLH105
Os01g0286900	Similar to Auxin-responsive protein IAA31 (Indoleacetic acid-induced protein 31). (Os01t0286900-01)	chr01:10312342..10314826	
Os01g0566100	Conserved hypothetical protein. (Os01t0566100-01);Ortholog of Arabidopsis ELF3, Regulation of flowering time (Os01t0566100-02)	chr01:21637031..21643111	OsELF3.2, ELF3_chr.1, OsELF3-2
Os01g0626400	WRKY transcription factor, Control of flowering time and plant height (Os01t0626400-01);WRKY transcription factor, Splicing variant (Os01t0626400-02)	chr01:25009514..25012233	WRKY11, OsWRKY11.2
Os01g0675700	Similar to Auxin-responsive protein IAA14 (Indoleacetic acid-induced protein 14) (SOLITARY-ROOT protein). (Os01t0675700-01);Similar to Auxin-responsive protein IAA14 (Indoleacetic acid-induced protein 14) (SOLITARY-ROOT protein). (Os01t0675700-02)	chr01:27777432..27780995	
Os01g0713600	B3 DNA-binding domain-containing transcription factor, Flowering time regulation, Repression of flowering activator Ehd1 and its downstream genes by binding to the promoter of the Ehd1 gene (Os01t0713600-01)	chr01:29637792..29642862	OsLFL1
Os01g0741900	Auxin-responsive protein, Drought tolerance, Control of tiller outgrowth (Os01t0741900-01)	chr01:30975765..30979337	IAA6, OsIAA6
Os01g0764800	Indole-3-acetic acid (IAA)-amido synthetase, Disease resistance, Abiotic stress tolerance (Os01t0764800-01)	chr01:32221378..32225121	GH3-2, OsGH3-2
Os01g0846450	Hypothetical conserved gene. (Os01t0846450-01);Homolog of Arabidopsis thaliana HEN1 suppressor 1, Heading date (Os01t0846450-02)	chr01:36353072..36358534	HESO1
Os01g0934300	Similar to Flowering-time protein isoform alpha. (Os01t0934300-01);Similar to Flowering-time protein isoform alpha. (Os01t0934300-02);Similar to Flowering-time protein isoform alpha. (Os01t0934300-03);Similar to Flowering-time protein isoform alpha. (Os01t0934300-04)	chr01:40984511..40992954	
Os01g0949400	Phytochromobilin (PΦB) synthase, Phytochrome chromophore biosynthesis, Photoperiodic response (Os01t0949400-01);Similar to elongated mesocotyl1. (Os01t0949400-02)	chr01:41822269..41825087	Se13, OsHY2
Os01g0971800	Transcription factor with a GARP DNA-binding domain, Photoperiodic control of flowering time, Clock associated-component (Os01t0971800-01)	chr01:42874273..42875515	OsPCL1
Os02g0209100	Similar to Coatomer beta' subunit (Beta'-coat protein) (Beta'-COP) (p102). (Os02t0209100-01)	chr02:6131817..6138156	
Os02g0228900	Similar to Auxin-responsive protein IAA18 (Indoleacetic acid-induced protein 18). (Os02t0228900-01)	chr02:7245057..7248588	
Os02g0232300	Similar to ZCN13. (Os02t0232300-00)	chr02:7485426..7487005	
Os02g0290500	Phosphatidylinositol 3-/4-kinase (PI3/4K) family protein, Ubiquitin-like domain kinase, Control of flowering time (Os02t0290500-01);Similar to phosphatidylinositol 3- and 4-kinase family protein. (Os02t0290500-02)	chr02:10994252..10997615	OsUbDKγ4, OsUbDKg4
Os02g0531600	TFL1/CEN ortholog, Phosphatidylethanolamine-binding protein, PEBP family protein, Control of the phase transition of meristem, Control of flowering time (Os02t0531600-01)	chr02:19567046..19568968	RCN2
Os02g0573200	Similar to Cryptochrome 1. (Os02t0573200-01);Cryptochrome 1a, blue light photoreceptor, Regulation of blue light-mediated de-etiolation, Blue light-dependent inhibition of coleoptile and leaf elongation (Os02t0573200-02)	chr02:21976544..21980296	OsCRY1a, OsCRY1, CRY1a, CRY2
Os02g0618200	Circadian-associated rice pseudo response regulator, Control of flowering time (Os02t0618200-01)	chr02:24569489..24572391	OsPRR1, OsTOC1, OsPRR1/OsTOC1
Os02g0625000	Cryptochrome 2, blue light photoreceptor, Promotion of flowering time (Os02t0625000-01);Similar to Cryptochrome 2. (Os02t0625000-02)	chr02:24915862..24922083	OsCRY2, CRY2
Os02g0723400	Similar to Isoform 2 of Auxin-responsive protein IAA8. (Os02t0723400-01);AUX/IAA protein family protein. (Os02t0723400-02)	chr02:30064358..30066172	
Os02g0771100	Ortholog of Arabidopsis CONSTITUTIVE PHOTOMORPHOGENIC1, Regulation of the onset of the adult phase, Regulation of vegetative-reproductive phase change and the time of flowering (Os02t0771100-01);Similar to CopI. (Os02t0771100-02);Similar to COP1 (Fragment). (Os02t0771100-03)	chr02:32528080..32533583	PPS, COP1
Os02g0793900	Ubiquitous nuclear protein, Regulation of photoperiodic flowering (Os02t0793900-01)	chr02:33734437..33737329	HDR1
Os02g0805100	Similar to Auxin-responsive protein IAA12 (Indoleacetic acid-induced protein 12) (BODENLOS protein). (Os02t0805100-01)	chr02:34348582..34349843	
Os02g0817600	AUX/IAA protein family protein. (Os02t0817600-01);Similar to Auxin-responsive protein IAA10. (Os02t0817600-02);AUX/IAA protein family protein. (Os02t0817600-03)	chr02:35073371..35075799	
Os03g0112700	Zinc finger CCCH domain-containing protein (Os03t0112700-01);CCCH-type zinc finger protein, Photoperiodic control of flowering time (Os03t0112700-02)	chr03:717447..720837	EHD4, OsC3H20, C3H20, Ehd4
Os03g0151300	Similar to JmjC domain containing protein, expressed. (Os03t0151300-01);JmjC domain-containing protein with a zinc-finger (ZnF) domain, Demethylase of H3K4 methylation, Suppression of flowering time under long day conditions (Os03t0151300-02)	chr03:2833113..2841685	Se14
Os03g0169600	Dof (DNA binding with one finger) transcription factor, Regulation of Flowering time, Brassinosteroid (BR) signaling to modulate plant architecture (Os03t0169600-01)	chr03:3738868..3741611	RDD4, OsDof12, Dof12, OsDof-12, OsCDF1, OsCDF1/OsDOF12, OsDOF12, CDF1, DOF12
Os03g0227000	Similar to Coatomer gamma subunit (Gamma-coat protein) (Gamma-COP). (Os03t0227000-01)	chr03:6676410..6683840	
Os03g0251350	NUCLEAR FACTOR Y (NF-Y) transcription factor, Control of heading date (Os03t0251350-01)	chr03:7976995..7979371	OsHAP5C, OsNF-YC2
Os03g0284100	Similar to Two-component response regulator-like PRR73. (Os03t0284100-01);Similar to Two-component response regulator-like PRR73. (Os03t0284100-02);Similar to Two-component response regulator-like PRR73. (Os03t0284100-03);Circadian-associated rice pseudo response regulator, Control of flowering time (Os03t0284100-04)	chr03:9759666..9768689	OsPRR73
Os03g0309200	Similar to Phytochrome B. (Os03t0309200-01);Phytochrome B, Control of deetiolation and flowering, Transduction of the night break (NB) signal to inhibit flowering, Regulation of critical day length (Os03t0309200-02)	chr03:11020131..11028178	PHYB, phyB
Os03g0391700	Helix-loop-helix DNA-binding domain containing protein. (Os03t0391700-01)	chr03:15685589..15689245	
Os03g0417700	Similar to Phytochrome P450. (Os03t0417700-01);Cytochrome P450 paralogue, Promotion of grain growth (Os03t0417700-02)	chr03:17340417..17342284	GL3.2

Os03g0445700	LATERAL ORGAN BOUNDARIES DOMAIN (LBD) protein, Transcriptional activator, Regulation of heading date and yield (Os03t0445700-01)	chr03:18935194..18936295	OsLBD37
Os03g0603100	Similar to Phytochrome P450. (Os03t0603100-00)	chr03:22568104..22568742	
Os03g0609500	LATERAL ORGAN BOUNDARIES DOMAIN (LBD) protein, Transcriptional activator, Regulation of heading date and yield (Os03t0609500-01)	chr03:22976955..22979863	OsLBD38
Os03g0633500	A member of Aux/IAA family protein, Regulation of lateral root initiation (Os03t0633500-01)	chr03:24198649..24202021	IAA11, OsIAA11
Os03g0633800	Similar to IAA6 (Fragment). (Os03t0633800-01)	chr03:24212554..24213858	
Os03g0639300	Similar to Helix-loop-helix DNA-binding domain containing protein, expressed. (Os03t0639300-01)	chr03:24544511..24549550	
Os03g0685000	Ferredoxin-like protein, Photosynthetic rate, Heading date, Chloroplast development (Os03t0685000-01)	chr03:27309857..27312902	OsFdC2, OsHDY1
Os03g0693600	Glucosyltransferase, Mediation of auxin homeostasis, Regulation of growth and development (Os03t0693600-01)	chr03:27775340..27778427	OsIAGT1, OsIAAGLU
Os03g0711100	Member of the CONSTANS-like (COL) family, Flowering time repressor (Os03t0711100-01)	chr03:28686958..28689501	OsCOL10, OsBBX10, OsJ, OsCCT14
Os03g0719700	Similar to Phytochrome A. (Os03t0719700-01)	chr03:29167195..29173721	
Os03g0719800	Phytochrome A, Photoreceptor (Os03t0719800-01)	chr03:29168197..29176006	PHYA, phyA, PHYA* (phyA), PHYA*, Phya, PHY18, phy 18, OsPHYA
Os03g0742900	Auxin/indole-3-acetic acid protein, Control of aerenchyma and lateral root development (Os03t0742900-01);Similar to cDNA clone:006-206-C11, full insert sequence. (Os03t0742900-03)	chr03:30480720..30483289	IAA13
Os03g0752100	Phytochrome C, Control of deetiolation and flowering, Photoperception of continuous far-red light (Os03t0752100-01)	chr03:31004724..31009758	PHYC
Os03g0762000	Similar to Protein kinase CK2, alpha subunit. (Os03t0762000-01);Casein kinase II alpha subunit, Heading date, Photoperiod sensitivity (Os03t0762000-02)	chr03:31508813..31514460	HD6, Hd6, Hd6(t)*, OsCKA2, OSCKA2
Os03g0773300	Protein kinase, core domain containing protein. (Os03t0773300-01);Similar to wound and phytochrome signaling involved receptor like kinase. (Os03t0773300-02)	chr03:32043966..32048393	
Os03g0782500	Phytochrome-interacting factor-like bHLH protein, Stress-responsive transcription factor, Regulator of reduced internode elongation under drought conditions (Os03t0782500-01)	chr03:32432551..32435839	PIL13, OsPIL1, OsPIL13, OsbHLH152
Os03g0793500	Casein kinase I, Photoperiodic control of flowering time, Long-day repression (Os03t0793500-01);Similar to Casein kinase-like protein. (Os03t0793500-02)	chr03:32999502..33006898	HD16, Hd16, EL1, CK1
Os03g0797800	AUX/IAA protein family protein. (Os03t0797800-01)	chr03:33246011..33247318	
Os03g0815700	K Homology domain containing protein, Nuclear RNA/DNA binding protein of the STAR (Signal Transduction and Activation of RNA) family, Flowering time control (Os03t0815700-01)	chr03:34186437..34190737	Spin1, SPIN1
Os03g0836800	Similar to IAA-amino acid hydrolase 1 (EC 3.5.1.-). (Os03t0836800-01)	chr03:35161307..35163174	
Os04g0166600	Homolog of yeast SAP30 (sin3-associated polypeptide 30), Regulation of flowering time, Mediation of periodic histone deacetylation (Os04t0166600-01)	chr04:4543619..4547486	OsSFL1
Os04g0429100	Histone H3 lysine 36-specific methyltransferase, Control of flowering time (Os04t0429100-01)	chr04:21258050..21262812	OsSET19, SDG708
Os04g0452100	Cryptochrome 1, blue light photoreceptor (Os04t0452100-01);Cryptochrome 1b, blue light photoreceptor, Regulation of blue light-mediated de-etiolation, Blue light-dependent inhibition of coleoptile and leaf elongation (Os04t0452100-02)	chr04:22549901..22555038	OsCRY1b, CRY1b, CRY1
Os04g0460600	Similar to H0219H12.10 protein. (Os04t0460600-01);NAC transcription factor, Regulation of shoot branching, plant height and flowering time (Os04t0460600-02)	chr04:22994561..22996624	NAC2, OsNAC2, ONAC004, ONAC4, ONAC034, ONAC34, ONAC058, ONAC58, OMTN2, Ostil1, OsNAC2/ONAC004, OsORE1.2
Os04g0488400	Similar to FLOWERING LOCUS T protein. (Os04t0488400-01)	chr04:24391459..24395755	
Os04g0569100	Similar to OCL1 homeobox protein. (Os04t0569100-01);Homeodomain-leucine zipper IV protein, GL2-type homeobox protein, Transcription factor, Promotion of flowering time preferentially under long days, Positive regulation of cuticular wax biosynthesis, Drought stress response (Os04t0569100-02)	chr04:28600699..28607038	ROC4, Roc4
Os04g0618600	Similar to Long hypocotyl in far-red 1 (bHLH-like protein HFR1) (Reduced phytochrome signaling) (Basic helix-loop-helix FBI1 protein) (Basic helix-loop-helix protein 26) (bHLH26) (AtbHLH026) (Reduced sensitivity to far-red light). (Os04t0618600-01)	chr04:31416378..31420095	
Os04g0648800	C3HC4 RING domain-containing E3 ubiquitin ligase, Modulation of heading date by physically interacting with Hd1 (Os04t0648800-01);Similar to Zinc finger, C3HC4 type family protein. (Os04t0648800-02)	chr04:33022835..33028358	OsHIP1, HIP1, HAF1, OsHAF1
Os04g0665200	SABATH family protein, IAA homeostasis (Os04t0665200-01)	chr04:33950246..33952563	IAMT1, OsIAMT1, OsSABATH4
Os05g0139100	Typical DNA-binding bHLH protein, Negative regulation of grain length and weight (Os05t0139100-01)	chr05:2246835..2248876	APG
Os05g0169300	Tryptophan aminotransferase, Indole-3-acetic acid (IAA) biosynthesis, Grain development (Os05t0169300-01);Similar to Tryptophan aminotransferase. (Os05t0169300-02)	chr05:4152905..4155880	TAR1, OsTAR1, FBL
Os05g0178600	Similar to Auxin-responsive protein (Aux/IAA) (Fragment). (Os05t0178600-01)	chr05:4681468..4684497	
Os05g0181200	Similar to Phytochrome P450-like protein. (Os05t0181200-01)	chr05:4885421..4887144	
Os05g0186900	Similar to Auxin-responsive protein IAA16. (Os05t0186900-01)	chr05:5329989..5331482	
Os05g0230700	Similar to Auxin-responsive protein IAA17. (Os05t0230700-01);Similar to Auxin-responsive protein IAA17. (Os05t0230700-02);Similar to Auxin-responsive protein IAA17. (Os05t0230700-03)	chr05:7950915..7954374	
Os05g0310800	Similar to Coatomer delta subunit (Delta-coat protein) (Delta-COP). (Os05t0310800-01)	chr05:14181444..14186266	
Os05g0311000	Clathrin adaptor, mu subunit, C-terminal domain containing protein. (Os05t0311000-01);Similar to delta-COP. (Os05t0311000-02)	chr05:14191542..14197286	
Os05g0317900	Catharanthus roseus receptor-like kinase1-like kinase, Regulation of flowering time signaling pathway and/or circadian rhythms (Os05t0317900-01)	chr05:14725926..14735830	OsCrRLK1L2
Os05g0494100	Histone-like transcription factor, Transcriptional repressor, Repressor of heading date, Inhibition of flowering under long-day condition (Os05t0494100-01)	chr05:24275966..24280288	DRAP2, OsDrAp2, OsHAPL1
Os05g0518000	Homolog of Hd3a, Promotion of flowering, Drought tolerance (Os05t0518000-01)	chr05:25667364..25669371	OsFTL10
Os05g0523300	Similar to IAA8 (Fragment). (Os05t0523300-01)	chr05:26052916..26055714	
Os05g0541400	Atypical bHLH transcription factor, Regulation of flowering time (Os05t0541400-01)	chr05:26879444..26881353	OsLF
Os05g0559400	Similar to IAA11 (Fragment). (Os05t0559400-01);Similar to IAA11 (Fragment). (Os05t0559400-02);Similar to IAA11 (Fragment). (Os05t0559400-03)	chr05:27846193..27850391	
Os05g0586200	Gretchen hagen 3 (GH3) family protein, Phytochrome, Jasmonyl-L-isoleucine synthase, Jasmonate signaling, Wound response, Response to blast infection (Os05t0586200-01)	chr05:29200200..29205715	OsGH3-5, OsGH3.5, OsJAR1, JAR1
Os06g0142600	Homolog of the Arabidopsis EARLY FLOWERING 3 protein, Photoperiodic flowering, Short-day/long-day promotion (Os06t0142600-01)	chr06:2234581..2239058	HD3B, Hd17, EF7, Ef7, OsELF3-1, OsELF3, ELF3_chr.6
Os06g0143900	Similar to Coatomer protein complex, beta prime; beta'-COP protein. (Os06t0143900-01);Similar to Coatomer complex subunit. (Os06t0143900-02);Similar to Coatomer complex subunit. (Os06t0143900-03)	chr06:2320837..2329651	
Os06g0157500	Florigen, Flowering time, Long-day promotion (Os06t0157500-01)	chr06:2926823..2928474	RFT1, FT-L 3, OsFTL3, FT-L3, RFT
Os06g0157700	Florigen, Heading date (Os06t0157700-01)	chr06:2940004..2942452	HD3A, Hd3a, FT, qHD3(t)*, FT-L 2, OsFTL2, FT-L2, FTL2
Os06g0166500	Similar to Auxin-responsive protein IAA20 (Indoleacetic acid-induced protein 20). (Os06t0166500-01)	chr06:3342464..3346318	
Os06g0184500	A member of the NPY gene family, Inflorescence development (Os06t0184500-01)	chr06:4229401..4233324	OsNPY2
Os06g0275000	Zinc finger protein, Heading date (Os06t0275000-01)	chr06:9336376..9338569	SE1, Se1(Lm,Lf,Rs,Fl), se(t), Hd1, HD1, Fl, Lm, Se1, K, Rs, Lf, Se-1, Hd1(t), qHD1(t), OsA, OsBBX18, Hd1/OsA, OsCCT21

Os06g0335500	AUX/IAA protein family protein. (Os06t0335500-01);Similar to Auxin-responsive protein IAA21. (Os06t0335500-02)	chr06:13345347..13349055	
Os06g0355300	Similar to Auxin-responsive protein IAA22. (Os06t0355300-01)	chr06:14577762..14580200	
Os06g0552900	Similar to SP3D. (Os06t0552900-00)	chr06:20971660..20974252	
Os06g0597000	Auxin/Indole-3-Acetic Acid (Aux/IAA) gene family member, Postembryonic maintenance of QC (quiescent center), Development of roots and shoots (Os06t0597000-01);Similar to Auxin-responsive protein IAA23. (Os06t0597000-02)	chr06:23501973..23503491	OsIAA23
Os06g0603000	Heme-oxygenase, Phytochrome chromophore biosynthesis, Nitric oxide- and auxin-induced lateral root formation (Os06t0603000-01)	chr06:23853783..23858023	SE5, Se5, OsHO1, OsHO-1, OsHY1, HO-1, HO1, OsSE5, PE-1
Os06g0614000	A member of the family of multiple C2 domain and transmembrane region proteins (MCTPs), Mediation of flowering time, Regulation of florigen transport (Os06t0614000-01)	chr06:24555313..24557973	OsFTIP1
Os06g0623700	Protein with indole-3-acetic acid (IAA)-glucose hydrolase activity, Regulation of grain length and weight (Os06t0623700-01)	chr06:25093242..25094294	TGW6, tgw6
Os06g0661800	Similar to Cryptochrome DASH, chloroplastic/mitochondrial. (Os06t0661800-01);Similar to Cryptochrome dash (Fragment). (Os06t0661800-02)	chr06:27275526..27278844	
Os06g0691400	Similar to IAA-amino acid conjugate hydrolase-like protein (Fragment). (Os06t0691400-01)	chr06:28831648..28837128	
Os06g0728700	Homeodomain-like containing protein. (Os06t0728700-01)	chr06:31025972..31028984	
Os07g0143200	Phytochrome-interacting bHLH factor, Cross-talk between light and stress signaling (Os07t0143200-01);Cold-induced alternative splicing variant of OsPIF14 (Os07t0143200-02)	chr07:2207362..2209018	OsPIF14, OsPIF14β, OsPIF14b, PIL14
Os07g0182200	Similar to Phytochrome-interacting factor 3 (Phytochrome-associated protein 3) (Basic helix-loop-helix protein 8) (bHLH8) (AtbHLH008). (Os07t0182200-01)	chr07:4339902..4342192	
Os07g0182400	AUX/IAA protein family protein. (Os07t0182400-01)	chr07:4355211..4356572	
Os07g0201100	Similar to Coatomer gamma subunit (Gamma-coat protein) (Gamma-COP). (Os07t0201100-01)	chr07:5452173..5459549	
Os07g0247100	Chloroplast-localised putative leucine carboxyl methyltransferase (LCMT), O-methyltransferase, Regulation of jasmonate (JA)- and brassinosteroid (BR)-mediated growth and defence responses, Leaf senescence, heading date and grain production via melatonin biosynthesis (Os07t0247100-01)	chr07:8194024..8199881	LLB, OsLLB, LCMT, OsMTS1, pls3
Os07g0249700	Similar to IAA-amino acid hydrolase ILR1-like 7. (Os07t0249700-01)	chr07:8321651..8325048	
Os07g0249800	Similar to IAA-amino acid hydrolase ILR1-like 8. (Os07t0249800-01)	chr07:8326644..8329267	
Os07g0261200	CCT(CONSTANS, CONSTANS-LIKE, and TIMING OF CHLOROPHYLL A/B BINDING1) domain protein, Heading date, Long-day repression, Regulator of growth, development, and stress-response (Os07t0261200-01)	chr07:9152377..9155030	GHD7, Ghd2, Ghd7, OsCCT26, OsCMF8, OsI
Os07g0592600	Indole-3-acetic acid (IAA)-amido synthetase, Auxin signaling and defense signaling in a pathogen-nonspecific manner (Os07t0592600-01)	chr07:24149718..24152074	GH3-8, OsGH3-8, OsMGH3, OsGH3.8
Os07g0695100	Pseudo response regulator, Heading date, Long-day repression (Os07t0695100-01);Similar to Two-component response regulator-like PRR37. (Os07t0695100-02)	chr07:29616705..29629215	Hd2, OsPRR37, PRR37, Prr4, OsPRR4, DTH7, OsCCT28, Ghd7.1, EH7-2
Os08g0105000	Homeodomain (PHD) transcriptional regulator, Flowering promoter (Os08t0105000-01);Zinc finger, RING/FYVE/PHD-type domain containing protein. (Os08t0105000-02);Similar to PHD-finger family protein. (Os08t0105000-03)	chr08:272854..276978	EHD3
Os08g0109400	AUX/IAA protein family protein. (Os08t0109400-01)	chr08:468151..473510	
Os08g0143400	SWIRM and amine oxidase domain-containing protein, Homolog of Arabidopsis FLOWERING LOCUS D (FLD), Control of flowering time (Os08t0143400-01);Similar to cDNA clone:J013128B21, full insert sequence. (Os08t0143400-02)	chr08:2385537..2389276	Hd18, OsHDMA703, HDMA703
Os08g0174500	Putative HAP3 subunit of the CCAAT box-binding transcription factor, Flowering time, Short-day promotion, Long-day repression (Os08t0174500-01);Similar to Heading date 5. (Os08t0174500-02)	chr08:4332106..4334829	HD5, Hd5(t)*, Hd5, qHD5, HAP3H, OsHAP3H, NF-YB, CBF-A, DTH8, qHD5(t)*, Ghd8, Ghd8/DTH8, Lhd1, NF-YB11, OsNF-YB11
Os08g0368000	Similar to Coatomer delta subunit (Delta-coat protein) (Delta-COP). (Os08t0368000-01)	chr08:17121810..17127723	
Os08g0496500	NUCLEAR FACTOR Y (NF-Y) transcription factor, Control of heading date (Os08t0496500-01)	chr08:24517598..24518559	OsHAP5D, OsNF-YC6
Os09g0123200	FCA-like protein, FCA gamma protein, Flowering time related protein involved in autonomous flowering pathway (Os09t0123200-01);Similar to Flowering time control protein isoform OsFCA-1. (Os09t0123200-02)	chr09:1790628..1799552	OsFCA
Os09g0134500	Hypothetical conserved gene. (Os09t0134500-01);Trithorax group protein, Regulation of flowering time (Os09t0134500-02)	chr09:2601653..2615934	OsTrx1, SDG723, OsSET33
Os09g0299000	Similar to CDPK substrate protein 1. (Os09t0299000-01);G2-like family transcription factor, Negative regulation of flowering time (Os09t0299000-02)	chr09:7316075..7322324	OsPHL3
Os09g0306700	Mediator subunit 25, Regulation of brassinosteroid (BR) signaling and plant architecture (Os09t0306700-01)	chr09:7914083..7925334	OsMED25
Os09g0306800	Polycomb group protein, Supressor of Zeste(12) homolog, Component of the polycomb repression complex 2 (PRC2), Control of flowering time (Os09t0306800-01)	chr09:7925628..7938161	OsEMF2b
Os09g0513500	Similar to ZCN24. (Os09t0513500-00)	chr09:19980206..19986378	
Os09g0527700	Similar to Auxin-induced protein IAA4. (Os09t0527700-01)	chr09:20641465..20643139	
Os09g0532400	Circadian-associated rice pseudo response regulator, Control of flowering time (Os09t0532400-01);Signal transduction response regulator, receiver region domain containing protein. (Os09t0532400-02)	chr09:20885173..20889792	OsPRR95
Os09g0560900	C2H2-type zinc finger transcription factor, Promotion of flowering (Os09t0560900-01);C2H2 zinc finger protein, Transcription factor, Regulation of flowering time (Os09t0560900-02)	chr09:22287174..22291746	SIP1
Os10g0397400	Dim/dwf1 protein, Cell elongation protein DIMINUTO/Dwarf1, Brassinosteroid (BR) biosynthesis (Os10t0397400-01);FAD-linked oxidoreductase protein, Brassinosteroid (BR) biosynthesis, Regulation of panicle and grain development, Regulation of heading date and chlorophyll biosynthesis (Os10t0397400-02)	chr10:13359545..13363225	BRD2, LTBSG1, LHDD10
Os10g0419200	Cys2/His2-type zinc finger transcription factor, Heading date (Os10t0419200-01)	chr10:14739603..14743098	Ehd2, RID1, OsID1, OsId1, RID1/OsID1/Ehd2
Os10g0463400	B-type response regulator, Floral inducer to promote short-day (SD) flowering pathway (Os10t0463400-01)	chr10:17076098..17081344	EF1, Ef1 (Ef2), Ef2, Ef1, Ehd1, Eh1(t)*, EF1, ef1, qEHD-10-1(t) (qEhd1), OsRR30, OsRR22
Os10g0565600	E3 ligases of H2Bub1, C3HC4-type RING finger protein, Transcriptional regulation of anther development, Regulaton of flowering time by affecting histone H2B monoubiquitination (Os10t0565600-01)	chr10:22388699..22395755	FRRP1, OsHUB2, HUB2
Os11g0152500	Putative phosphatidylethanolamine-binding protein, Rice TFL1/CEN homolog, Control of inflorescence architecture, Repression of flowering (Os11t0152500-01)	chr11:2452773..2454103	RCN1, FDR2
Os11g0157600	Circadian-associated rice pseudo response regulator, Control of flowering time (Os11t0157600-01)	chr11:2789002..2793728	OsPRR59
Os11g0187200	GATA zinc finger family protein, Control of heading date, plant height and panicle length (Os11t0187200-01)	chr11:4432745..4434088	OsGATA28
Os11g0221000	Aux/IAA_ARF_dimerisation domain containing protein. (Os11t0221000-01)	chr11:6335893..6342309	
Os11g0221200	Putative auxin-responsive protein IAA28. (Os11t0221200-00)	chr11:6348483..6349758	
Os11g0221300	AUX/IAA protein family protein. (Os11t0221300-00)	chr11:6356838..6358201	
Os11g0250000	Similar to RNA recognition motif family protein, expressed. (Os11t0250000-01);Heterogeneous nuclear ribonucleoprotein (hnRNP) R-type protein, Flowering time regulation (Os11t0250000-02)	chr11:8100740..8105413	RBS1, Rbs1
Os11g0293800	Similar to ZCN17. (Os11t0293800-00)	chr11:10732822..10734815	

Os11g0525200	Obtusifoliol 14α-demethylase, Phytosterol biosynthesis, Regulation of heading date and seed production (Os11t0525200-01);Similar to Cytochrome P450 51 (EC 1.14.13.70) (CYPLI) (P450-LIA1) (Obtusifoliol 14-alpha demethylase) (Fragment). (Os11t0525200-02)	chr11:19048566..19052876	OsCYP51G1
Os11g0528700	Indole-3-acetic acid (IAA)-amido synthetase, Plant architecture establishment, Drought tolerance (Os11t0528700-01);Indole-3-acetic acid (IAA)-amido synthetase (Os11t0528700-02)	chr11:19184337..19190125	TLD2
Os11g0545600	Reader protein of trimethylated histone H3 lysine 4 (H3K4me3) and histone H3 lysine 36 (H3K36me3), Brassinosteroid-regulated growth, Flowering time control (Os11t0545600-01);Similar to H0102C09.3 protein. (Os11t0545600-02);MRG family protein. (Os11t0545600-03)	chr11:20098066..20106820	MRG702
Os11g0706200	GRAS protein, Regulation of heading date and yield (Os11t0706200-01)	chr11:28899015..28900796	DHD1
Os12g0232300	Similar to ZCN20. (Os12t0232300-00)	chr12:7235000..7237115	
Os12g0601300	Similar to Auxin-responsive protein (Aux/IAA) (Fragment). (Os12t0601300-01)	chr12:25310893..25316150	
Os12g0601400	Similar to Auxin-responsive protein (Aux/IAA) (Fragment). (Os12t0601400-01)	chr12:25325555..25327111	
Os12g0610200	Basic helix-loop-helix transcription factor, Regulation of grain size (Os12t0610200-01)	chr12:25768808..25773052	OsPIL11, OsbHLH102
